# Determining the association between different living arrangements and depressive symptoms among over-65-year-old people: The moderating role of outdoor activities

**DOI:** 10.3389/fpubh.2022.954416

**Published:** 2022-08-04

**Authors:** Rixiang Xu, Yulian Liu, Tingyu Mu, Yaping Ye, Caiming Xu

**Affiliations:** ^1^School of Humanities and Management, Zhejiang Chinese Medical University, Hangzhou, China; ^2^Ningbo Municipal Hospital of TCM Affiliated Hospital of Zhejiang Chinese Medical University, Ningbo, China; ^3^School of Nursing, Zhejiang Chinese Medical University, Hangzhou, China; ^4^School of Law, Zhejiang University City College, Hangzhou, China

**Keywords:** depression, living arrangement, eldercare institutions, aging population, outdoor activities

## Abstract

**Background:**

China is presently facing the challenge of meeting enormous health demands because of its rapidly aging society. Enrolling older persons in eldercare institutions is a helpful alternative for relieving family caregivers and promoting healthy aging. However, changes in the living environment may negatively affect the mental health of the elderly.

**Objective:**

To explore the association between different living arrangements and depressive symptoms among over-65-year-old people in China and the moderating role of outdoor activities.

**Method:**

The 2018 wave of the Chinese Longitudinal Healthy Longevity Survey (CLHLS) used a mixed sampling method to collect the health and demographic information of 15,874 older adults over 65 years from 23 provinces in China. After considering this study's inclusion and exclusion criteria, the final sample comprised 12,200 participants. The participants' risk of depressive symptoms was assessed using the 10-item Center for Epidemiologic Studies Depression Scale (CESD-10). The potential association between the two elements was tested using a regression model.

**Result:**

This study's findings suggested a significant relationship between depressive symptoms and living arrangements (*P* < 0.001). Participants living alone and those living in eldercare institutions had 1.26-times (95%CI: 1.10–1.44) and 1.39-times (95%CI: 1.09–1.77) higher risks of depressive symptoms, respectively, than those living with household members. Outdoor activities play a moderating role between different living arrangements and depressive symptoms. Among participants who engaged in outdoor activities, no significant difference was observed in the risk of depressive symptoms between those living in eldercare institutions and those living with household members (adjusted odds ratio = 1.15, 95%CI = 0.81–1.64, *P* = 0.426).

**Conclusion:**

The high risk of depressive symptoms among older Chinese people living alone or in eldercare institutions requires considerable attention. The evidence from this study suggests that older people living alone and those living in eldercare institutions should regularly engage in appropriate outdoor activities.

## Introduction

Population aging (and its adverse effects) is becoming a global challenge (1). As in Japan, South Korea, and some European countries, population aging in China has emerged as an increasingly crucial social issue in the last decade. According to the National Bureau of Statistics of China, the number of older people aged over 65 years in China reached 200.56 million (14.2% of the total population) by the end of 2021, indicating that China has become a rapidly aging society (2). The main contributors to this issue are the declining birth rate and the increasing life expectancy (3). The immediate threat of population aging to society is increasing labor and health costs due to chronic age-related conditions. Empirical evidence suggests that age-related diseases, especially among people aged over 65 years, account for over half of the global burden among adults (4). Population aging has also resulted in a social burden in China, potentially contributing to 92.8 million disability-adjusted life-years (DALYs) between 1997 and 2017 (5). Therefore, given that the current population aging trend is almost irreversible, each government's effort focuses on improving health outcomes in the aging population.

Depression is defined as a group of symptoms forming a syndrome and causing functional impairment (6). Depression consists of three main subtypes, including emotional symptoms, neurovegetative symptoms, and neurocognitive symptoms; depressed mood and anhedonia are the fundamental symptoms of depression (6). Depression negatively contribute to the quality of life in aging populations (7). Not only can they considerably reduce each individual's well-being, especially based on the declining physical function in older people, they are also a high-risk factor for many diseases or adverse events (such as suicide, pain, chronic diseases, disability, among others), resulting in more DALYs and years of life lost (YLLs) (8–10). Unfortunately, the prevalence of depressive symptoms among elderly Chinese people was as high as 35.19% (11). Therefore, the research focusing on promoting the mental health of the elderly holds great value at both the social and individual levels.

At present, expanding the industrial scale of eldercare institutions is an essential measure to achieve positive aging and ease the economic burden on society in China (12). Living in eldercare institutions is deemed appropriate behavior because the elderly can benefit from the professional services provided by licensed caregiving staff (13), and family caregivers can be relieved by enrolling older persons in elder care institutions, especially in the era of the post-one-child policy (a family planning measure aimed to control the rapidly growing population). These factors will encourage an increasing population of elderly Chinese individuals to live in eldercare institutions. However, a high prevalence of depressive symptoms was observed among older people living in eldercare institutions (14). Therefore, this study determined the association between depressive symptoms and living arrangements, and explore the moderating role of outdoor activities between depressive symptoms and different living arrangements.

This research framework was designed using the environment-stress theory in environmental psychology (15). This theory states that changes in the living environment (based on how an individual perceives the environment, such as atmosphere, light, and color, among others) will affect an individual's emotional, behavioral, and physiological reactions. Positive emotional reactions in individuals are influenced by elements such as bright light, fresh air, and green spaces in outdoor activities (16–18). Furthermore, individual participation in outdoor activities boosts physical activity and social interaction. Based on the research experience in molecular biology, the serotonin hypothesis is one of the most popular causal hypotheses for depression, suggesting that depression is caused by lower concentrations of the serotonin neurotransmitter (19). Appropriate levels of physical activity can significantly increase the extracellular concentration of serotonin (20). Moreover, the evolutionary theory of loneliness contends that social interaction can effectively lower the increased physical and mental health risks caused by loneliness (21). Therefore, a national-level survey database was selected in this study to examine the differences in the risk of depressive symptoms among the elderly with different living arrangements and whether outdoor activities play a moderating role.

## Methods

### Study design and sample

This study's data were sourced from the Chinese Longitudinal Healthy Longevity Survey (CLHLS), which was conducted by the National School of Development at Peking University. The nationwide community-based prospective longitudinal study began in 1998, and examinations are conducted every 2–3 years (the latest wave was conducted around 2018). It aims to provide representative data for identifying the determinants of longevity. New participants are registered during the follow-up to reduce the attrition caused by death and loss to follow-up. The CLHLS (2018) was conducted in randomly selected counties and cities in 23 out of 31 provinces in Mainland China (including Beijing, Tianjin, Hebei, Shanxi, Liaoning, Jilin, Heilongjiang, Shanghai, Jiangsu, Zhejiang, Anhui, Fujian, Jiangxi, Shandong, Henan, Hubei, Hunan, Guangdong, Guangxi, Hainan, Chongqing, Sichuan, and Shaanxi). It focussed on the health status, quality of life, cognitive function, personality, psychological characteristics, daily activities, disease treatment, and medical expenses of the older Chinese people. The CLHLS was initially designed to facilitate global comparative analysis, and its survey instruments were translated into Chinese from the instruments of the Danish longevity survey (22). The CLHLS used a multistage cluster sampling method to include all consenting centenarians who live in selected areas. Next, other age groups of the same gender (65–80, 80s, and 90s) in the vicinity of the centenarian's place of residence (in the selected street or village, or in the selected city or county) were randomly invited for this study. Well-trained local Center for Disease Control investigators and university students were hired to conduct in-person interviews at the participants' residences. An earlier investigation reported a more detailed introduction to the CLHLS study design (23).

This study's data were sourced from the 2018 wave of the CLHLS. This wave of the CLHLS collected data from 15,874 participants, from October 2017 to July 2019. First, 95 participants younger than 65 years were excluded. Then, 3,403 participants were excluded because of inability or unwillingness to complete the ten-item Center for Epidemiologic Studies Depression Scale (CESD-10) or for answering data with apparent errors. In addition, 73 participants who failed to respond to questions regarding their living arrangements were also excluded. Finally, 12,200 eligible subjects were screened from 15,874 participants. From the data collected from all participants, a total of 12,461 participants completed the CESD-10 scale (a response rate of 78.50%) and 15,549 participants responded to the questions about their recent living arrangements (a response rate of 97.95%), and 15,619 participants reported outdoor activities in the survey (a response rate of 98.39%). The details of the eligible participants are described in the following section. All participants provided written informed consent at the time of participation, and the CLHLS data collection (IRB00001052–13074) was approved by the Biomedical Ethics Review Committee of Peking University.

### Measures

#### Depressive symptoms (dependent variable)

The CESD-10 was used to assess the participants' risk of depressive symptoms and was added to the 2018 wave of the CLHLS questionnaire for the first time. This scale was compiled by Anderson and used after being translated into Chinese (24). The Chinese version of the scale was proven to be well validated for the assessment of depression in the general Chinese elderly population (25). The scale contains eight forward-scoring and two reverse-scoring questions that measure the frequencies of the participant's negative feelings in the past week. A previous report provided specific information for each question (24). Each question has four response options with different frequencies: rarely or none of the time, some or a little of the time, occasionally or a moderate amount of the time, and most or all the time. Forward-scored questions are scored as 0–3 points according to the frequency from low to high, and 3–0 points are scored in the reverse direction. The total range of CESD-10 scores was 0–30, with scores ≥ 10 indicating significant depressive symptoms. The Cronbach's α of CES-D-10 was 0.730, indicating a reasonable reliability level of internal consistency. The Pearson correlation analysis revealed that each variable was significantly correlated with the total score of the scale (*P* < 0.01), indicating that the scale has reasonable content validity.

#### Living arrangements (independent variable)

In this study, living arrangement refers to the recent long-term living status: living with family members (spouse, parents, children, etc.), living alone (without the company of family members for a long time, including babysitters and sexual partners), and living in eldercare institutions (elderly center, elderly home, care home, etc.).

#### Outdoor activities (moderating variable)

Outdoor activities here refer to individuals leaving their familiar house to participate in positive activities, such as physical activity, social activities, playing chess, fishing, and traveling, among others. The questionnaire in the 2018 wave of the CLHLS included an item about the frequency of regular participation in outdoor activities (including Tai Chi, square dancing, socializing with friends, and other outdoor activities). The older people in China prefer these outdoor activities. There are five different frequencies under the question: There are five different frequencies under the question: (1) almost daily. (2) at least once weekly. (3) at least once monthly. (4) less than once monthly, and (5) never participate (scoring 4, 3, 2, 1, and 0 points, respectively)Scores <0 indicate participation in outdoor activities and scores equal to 0 indicate no participation in outdoor activities.

#### Covariates

The participants' demographic or socioeconomic characteristics (such as gender, age, living location, years of schooling, marital status, income, and hukou status) and health status (number of chronic diseases) were some of the control variables included in this study's analyses. This study included these covariates because they were described as the confounders for depressive symptoms in the previous studies or preliminary univariate analyses.

##### Age

Age as a risk factor for depressive symptoms in the Chinese elderly was reported in a previous study (26). Age in this study was divided into two groups: 65–80 and over 80 years old.

##### Hukou status

Hukou is a unique existence in China; it refers to the national household registration that Chinese governments have historically used to try to fix the population in a place geographically. The primary study reported a significant difference in the prevalence of depressive symptoms among residents with different hukou (27). In our study, Hukou was divided into agricultural and non-agricultural.

##### Marital status

Marital status was categorized as married and living with a spouse, married and living without a spouse, widowed, never married, or divorced. A national-level survey in China found that separation, widow, or divorce are risk factors for depressive symptoms (28).

##### Years of schooling

Since this database does not collect data on specific educational levels, it only includes each participant's years of schooling. Therefore, the years of schooling were divided into 0 years (illiterate), 1 to 9 years (elementary school or middle school), and over 10 years (high school and above), according to the length of different learning stages. Previous studies found that individuals with higher levels of education have a lower risk of depressive symptoms (28, 29).

##### chronic-diseases]Chronic diseases

A total of 13 chronic diseases were included, namely hypertension, dyslipidaemia, diabetes, cancer, hepatitis, heart attack, stroke & cerebrovascular disease, asthma & lung disease, Parkinson's disease, dementia, digestive disease, arthritis, and nephritis. Previous studies have reported a higher risk of depressive symptoms in patients with a higher number of chronic diseases (9).

##### Household income

Annual household income was classified as <10,000, 10,000–50,000, 50,000–100,000, and more than 100,000 CNY (1 USD≈6.62 CNY in 2018). A previous study found a negative correlation between income and depressive symptoms in the elderly in China (26).

##### Disability

The KATZ scale was used to estimate whether participants had difficulty in six activities: eating, dressing, bathing, transferring in and out of bed, using the toilet, and controlling urination and defecation. Respondents were identified as having a disability if they had difficulty completing one of the activities. In all individuals with a disability, difficulty completing 1–2 activities, 3–4 activities, and 5–6 activities were considered mild, moderate, and severe disability, respectively. Our previous study found that individuals with higher levels of disability were at greater risk of depressive symptoms (29). The Cronbach's αvalue was 0.908, indicating a reasonable reliability level.

### Data processing and analysis

The original data from the CLHLS database were exported in the SAV. format to Microsoft EXCEL 2016 for data screening and description. Before formally processing the data, an author (RXX) checked the legibility and completeness of the data. Any extraneous variables that must be controlled for and any potential problems in the data collection process were identified and avoided. Two other authors (YLL and TYM) independently performed data cleaning for outliers. Target data were transcoded and interpreted according to the CLHLS data coding guidelines (available at https://opendata.pku.edu.cn/dataverse/CHADS). All measurement data were recorded according to Supplementary Table 1 and imported into SPSS 25.0 (SPSS Inc., Chicago, IL, US) software for data analysis. Statistical analysis was performed using SPSS 25.0 (SPSS Inc., Chicago, IL, US). A chi-square test was applied to the association between independent, control, and moderator variables and depressive symptoms. A binary logistic regression adjusting for all confounding factors was conducted to compare the strength of the relationship between different living arrangements and depressive symptoms. Living arrangements and outdoor activities were sequentially included in the regression model to explore whether the association between living arrangements and depressive symptoms was affected after considering participation in outdoor activities. In addition, an interaction group for living arrangements and outdoor activities was introduced to determine whether outdoor activities moderated the independent and dependent variables. *P*-values <0.05 were considered statistically significant in this study.

## Results

### Sample characteristics

Table 1 presents the descriptive data for the sample and evaluates the odds risk (OR) value of depressive symptoms in control and independent variable groups. A total of 12,200 participants were included in this study, 46.5% of whom were male and 53.5% female, with a mean age of 83.39 ± 11.02 years. Most of the participants lived in rural areas (76.5%), had an agricultural hukou (70.8%), had <9 years of education (89.5%), and had lost their spouses (52.2%). Regarding the individual health status, 18.5% of respondents reported disabilities and 63.1% had one or more chronic diseases. Furthermore, 79.9, 16.8, and 3.3% of the respondents lived with household members, alone, and in eldercare institutions, respectively. Moreover, 30.4% of the older people did not engage in any outdoor activities, whereas 39.5% of the older people who participated in outdoor activities maintained the habit almost once daily.

**Table 1:** General characteristics of the participants by the occurrence of depressive symptoms and their unadjusted odds risk.

**Category**	**Total (%)**	**Depressive symptoms (** ***n*** **)**		**Crude OR (95%CI)**
		**No (9060)**	**Yes (3140)**	***P***	
**Age**				<0.001	
65–80	5297 (43.4)	4150	1147		1
80 <	6903 (56.6)	4910	1993		1.47 (1.35–1.60)^*^
**Gender**				<0.001	
Male	5667 (46.5)	4374	1293		1
Female	6533 (53.5)	4686	1847		1.33 (1.23–1.45)^*^
**Living location**				<0.001	
Urban	2871 (23.5)	2269	602		1
Rural	9329 (76.5)	6791	2538		1.41 (1.27–1.56)^*^
**Hukou status**				<0.001	
Non–agricultural	3560 (29.2)	2798	762		1
Agricultural	8623 (70.8)	6250	2373		1.39 (1.27–1.53)^*^
**Households income**				<0.001	
<10000	3016 (25.8)	2052	964		1
10000–50000	3721 (31.8)	2788	933		0.71 (0.64–0.79)^*^
50000–100000	2705 (23.1)	2045	660		0.69 (0.61–0.77)^*^
>100000	2257 (19.3)	1823	434		0.51 (0.45–0.58)^*^
**Marital status**				<0.001	
Married and living with spouse	5402 (44.7)	4283	1119		1
Married and living without spouse	224 (1.9)	174	50		1.10 (0.80–1.52)
Divorced	45 (0.4)	32	13		1.56 (0.81–2.97)
Widowed	6304 (52.2)	4431	1873		1.62 (1.49–1.76)^*^
Never married	103 (0.9)	56	47		3.21 (2.17–4.76)^*^
**Years of schooling**				<0.001	
0	4656 (44.3)	3225	1431		1
1–9	4758 (45.2)	3722	1036		0.63 (0.57–0.69)^*^
10–	1103 (10.5)	883	220		0.56 (0.48–0.66)^*^
**Disability**				<0.001	
No	9947 (81.5)	7608	2339		1
Mild	1341 (11.0)	930	411		1.44 (1.27–1.63)^*^
Moderate	490 (4.0)	294	196		2.17 (1.8–2.61)^*^
Severe	422 (3.5)	228	194		2.77 (2.27–3.37)^*^
**Number of chronic diseases**				<0.001	
0	4496 (36.9)	3439	1057		1
1	4056 (33.2)	3060	996		1.6 (0.96–1.17)
2	2072 (17.0)	1491	581		1.27 (1.13–1.43)^*^
3	928 (7.6)	662	266		1.31 (1.12–1.53)^*^
≥4	648 (5.3)	408	240		1.91 (1.61–2.28)^*^
**Living arrangement**				<0.001	
With household members	9747 (79.9)	7401	2346		1
Alone	2050 (16.8)	1394	656		1.49 (1.34–1.65)^*^
Elderly institution	403 (3.3)	265	138		1.64 (1.33–2.3)^*^
**Outdoor activities**				<0.001	
0	3711 (30.4)	2515	1196		1
1	849 (7.0)	591	258		0.92 (0.78–1.80)
2	754 (6.2)	548	206		0.79 (0.66–0.94)^*^
3	2064 (16.9)	1526	538		0.74 (0.66–0.84)^*^
4	4822 (39.5)	3880	942		0.51 (0.46–0.56)^*^

* *P <0.05; OR, odds rate*.

### Associations between living arrangements and depressive symptoms

Table 1 shows the results of the univariate analyses performed to determine the distributions of depressive symptoms by relevant covariates and independent variables. Participants with the following characteristics were significantly associated with a higher risk of depressive symptoms (*P* < 0.05): female (OR = 1.33, 95%CI = 1.23–1.45), over 80 years old (OR = 1.47, 95%CI = 1.35–1.6), living in rural areas (OR = 1.41, 95%CI = 1.27–1.56), with agricultural hukou (OR = 1.39, 95%CI = 1.27–1.53), widowed (OR = 1.62, 95%CI = 1.49–1.76), and never married (OR = 3.21, 95%CI = 2.17–4.76). In addition, participants with fewer years of schooling, a higher number of chronic diseases, lower annual household income, higher levels of disability, and lower frequency of outdoor activities had higher risks of depressive symptoms (*P* < 0.05). Table 1 also presents more details.

Table 2 presents the binary logistic regression analysis results testing the relationship between living arrangements and depressive symptoms. After controlling for confounders (Model 1), participants living alone had a 1.26-times higher risk of depressive symptoms than those living with household members (adjusted odds ratio [AOR] = 1.26, 95%CI = 1.10–1.44, *P* = 0.001), while for those living in eldercare institutions, this risk was 1.39-times higher (AOR = 1.39, 95%CI = 1.09–1.77, *P* = 0.008). In Model 2, the results demonstrated that outdoor activities could significantly reduce the risk of depressive symptoms in respondents (AOR = 0.90, 95%CI = 0.87–0.92, *P* < 0.001). Moreover, the introduction of the outdoor activities variable in the model revealed that the risk of depressive symptoms increased to 1.31 times (95%CI = 1.14–1.50, *P* < 0.001) and 1.41 times (95%CI = 1.11–1.80, *P* = 0.006) for respondents living alone and those living in eldercare institutions, respectively.

**Table 2:** Binary logistic regression for the association between living arrangements and depressive symptoms.

**Variables**	**Model 1**			**Model 2**		
	**AOR**	**95%CI**	***P***	**AOR**	**95%CI**	***P***
**Age**	1.07	0.96–1.20	0.224	1.01	0.90–1.14	0.825
**Hukou status**	1.13	0.95–1.34	0.168	1.12	0.95–1.33	0.178
**Living location**	1.24	1.03–1.48	0.021	1.25	1.05–1.50	0.015
**Gender**	1.05	0.95–1.17	0.352	1.06	0.95–1.18	0.294
**Household income**	0.84	0.80–0.89	<0.001	0.85	0.81–0.89	<0.001
**Marital status**	1.07	1.03–1.12	0.001	1.07	1.03–1.11	0.001
**Years of schooling**	0.87	0.79–0.95	0.002	0.88	0.81–0.97	0.006
**Number of chronic diseases**	1.22	1.17–1.27	<0.001	1.22	1.17–1.28	<0.001
**Disability**	1.37	1.29–1.46	<0.001	1.28	1.20–1.37	<0.001
**Living arrangements**						
With household members	Ref.	/	/	Ref.	/	/
Alone	1.26	1.10–1.44	0.001	1.31	1.14–1.50	<0.001
Elderly institution	1.39	1.09–1.77	0.008	1.41	1.11–1.80	0.006
**Outdoor activities**				0.90	0.87–0.92	<0.001

*Model 1, controlling for age, Hukou status, gender, living location, marital status, household income, years of schooling, number of chronic diseases, disability. Model 2, introducing outdoor activities variable on the basis of Mode 1. AOR, adjusted odds rate. Reference, compare to living with household members Ref., Reference*.

### Moderating effect of outdoor activities

**Figure 1:**
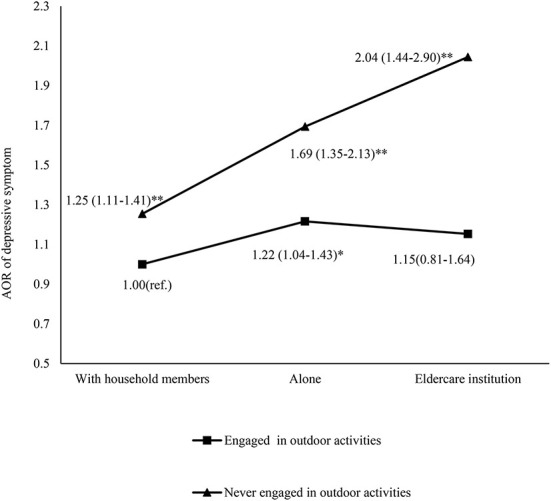
Interaction effects of outdoor activities and living arrangements in depression. (Logistic regression analysis controlling for age, Hukou status, gender, living location, marital status, household income, years of schooling, number of chronic diseases, and disability; * *P* < 0.05; ^**^P < 0.001; ref., reference; AOR, adjusted odds rate).

The results in Table 3 demonstrated the risk of depressive symptoms for the interaction groups of different living arrangements and outdoor activities. The binary regression model demonstrated a strong interaction effect between living arrangements and outdoor activities with regard to depressive symptoms. Overall, participants who never engaged in outdoor activities and lived in eldercare institutions were 2.04 times more likely to experience depressive symptoms (95%CI = 1.44–2.9, *P* < 0.001) than participants who had a habit of participating in outdoor activities and lived with household members, and those who never participated in outdoor activities and lived alone were 1.69 times more likely to experience depressive symptoms (95%CI = 1.35–2.13, *P* < 0.001). Figure 1 depicts the AOR values of depressive symptoms for different interaction groups after controlling for all covariates.

**Table 3:** Measuring the moderating effect of outdoor activities on living arrangements and depressive symptoms.

**Interaction group**	**Total (%)**	**B**	**SE**	**AOR**	**95%CI**	***P***
Living with household members × Have outdoor activities	6733 (55.19)	Ref.	Ref.	Ref.	Ref.	Ref.
Living with household members × No outdoor activities	3014 (24.70)	0.226	0.062	1.254	1.11–1.41	<0.001
Living alone × Have outdoor activities	1534 (12.57)	0.196	0.081	1.216	1.04–1.43	0.015
Living alone × No outdoor activities	516 (4.29)	0.527	0.116	1.694	1.35–2.13	<0.001
Living in elderly institution × Have outdoor activities	222 (1.82)	0.143	0.179	1.153	0.81–1.64	0.426
Living in elderly institution × No outdoor activities	181 (1.48)	0.715	0.178	2.044	1.44–2.90	<0.001

*controlling for age, Hukou status, gender, living location, marital status, household income, years of schooling, number of chronic diseases, disability. B, regression coefficient; SE, standard error; AOR, adjusted odds rate*.

## Discussion

This study used a national-level survey database to determine the relationship between living arrangements and depressive symptoms among 12,200 Chinese people aged over 65 years. It explored the moderating effect of outdoor activities on both these variables. The main findings of this study indicated that participants living alone and those living in eldercare institutions had a significantly higher risk of depressive symptoms than those living with household members, and outdoor activities were effective in eliminating the risk of depressive symptoms caused by different living arrangements. These findings provide compelling evidence for future government decision-making on promoting active and healthy aging.

This study was not the first to explore the relationship between living arrangements and depressive symptoms. Some empirical studies conducted in South Korea, Singapore, and Japan have discovered that living alone is an independent risk factor contributing to depressive symptoms (30–32), which was consistent with this study's findings. Some in-depth studies based on other perspectives compared the risk of depressive symptoms in elder adults living with different household members and revealed that living with children was negatively associated with elders' depressive symptoms (33, 34). All evidence suggested that living with household members was a protective factor against depressive symptoms. In addition, this study's results suggested a 34.2% prevalence of depressive symptoms among the elderly living in Chinese eldercare institutions. The mental health issues of this population have gradually aroused the interest of researchers in other countries and regions. Previous studies from Taiwan, Brazil, and the United States also reported a high prevalence of depression in this population (36–54.8%) (35–37). However, no empirical study had reported the risk of depressive symptoms among older adults choosing to enroll in eldercare institutions than other living arrangements, based on this study's rigorous literature search.

China currently faces a huge demand for elderly care services, which will gradually grow with the increasingly severe issue of population aging and the shortage of family caregivers (from 1982 to 2016, China had the peculiar 4-2-1 family structure where a couple could only have one child) (38). However, this study's results revealed that eldercare institutions are not a good alternative for the mental health of the elderly. In fact, the risk of depressive symptoms among the elderly living in eldercare institutions was 1.41 times higher than that among those living with household members. Differences in the depressive risk for different living arrangements could be explained or speculated for the following reasons. First, the traditional family-centered culture in China makes the elderly reluctant to leave their familiar house (even if they live alone) (39). A study conducted in the Zhejiang Province (China) discovered that only 3.8% of elderly adults were willing to enroll in an eldercare facility. This change in the living environment and their rejection psychology (maybe a stigma (13)) might be potential risk factors for depressive symptoms. Second, the noise pollution, crowding, and cramped indoor activity spaces of some eldercare institutions in China might contribute to the depressive symptoms of older adults. Some Chinese eldercare institutions face severe noise pollution and the lack of adequate indoor activity space because of irrational site selection and renovations from other-use buildings (40, 41). Moreover, several community eldercare institutions arrange for multiple people to live in one room. This lack of personal privacy and noisy space reduces the elderly people's quality of sleep. In previous investigations, these negative factors were found to be significantly associated with depressive symptoms (42, 43). Third, many elderly Chinese people regard living in an eldercare institution as “the last journey in their lives.” As the older adults around them die one after another, they feel like there is a countdown to their lives, making them pessimistic (44). Fourth, some eldercare institutions in China lack functional mental health services, and the caregivers working in these institutions hardly pay attention to the clients' mental health in their daily work. This deficiency is directly related to the shortage, old age, and low education level of the caregivers (45).

A key finding of this study suggested that outdoor activities, as a moderator between different living arrangements and depressive symptoms, reduced and eliminated the risk of depressive symptoms associated with older Chinese adults living alone and in eldercare institutions, respectively. Previous empirical studies have proven that outdoor activities were an effective measure for preventing depressive symptoms (46, 47). This study did not thoroughly explore the mechanisms of the moderating effect of outdoor activities between living arrangements and depression. However, the mechanism by which outdoor activities ameliorates depression is relatively clear. It can be viewed as a blend of strategies that include environmental factors (bright light, fresh air, green space, and ample space for activity), individual factors (increased physical activity), and social factors (increased socialization) (16, 17). This study included the types of outdoor activities that are generally preferred by the elderly in China, such as Tai Chi and square dancing, among others. Tai Chi is a way of improving immunity in traditional Chinese medicine. A systematic review and meta-analysis pooling 37 relevant randomized controlled trials showed that Tai Chi interventions exerted beneficial effects for various populations with regard to depressive symptoms (48). Tai Chi is a series of meditative movements. An earlier study demonstrated that meditation can alleviate several stress-related emotional symptoms by activating the parasympathetic nervous system (49). Similarly, square dancing reduced depressive symptoms in older adults, which has also been reported in previous intervention studies (50). Square dancing may reduce depression risk through socialization and exercises. Although this study advocated that elderly people living alone or in eldercare institutions should actively participate in outdoor activities to prevent depressive symptoms, it should also be remembered that each elder adult needs to select suitable activities according to their health status (because some patients are advised not to participate in outdoor activities).

This study had several limitations. First, it did not consider how long participants had lived alone or in eldercare institutions. This provision might interfere with this study's results as the risk of depressive symptoms may differ among participants with different event durations. Second, it did not categorize the outdoor activities the participants engaged in, which would affect the interpretation of the results. Third, the older people in this study living in eldercare intuitions accounted for only 3.3% of the total sample (403 cases), which may have biased the results. However, this study used a comprehensive analysis to avoid these biases.

### Practical implications

This study's results have practical implications for Chinese governments and organizers of future eldercare institutions in selecting the building sites and planning the internal organization and work content settings. First, the caregivers of the institutions should encourage and organize the elderly to participate in outdoor activities and cultivate their interests in some outdoor activities. Second, prioritizing the locations of eldercare institutions away from commercial areas and close to parks and squares can reduce the exposure of the elderly to noise pollution and facilitate their participation in outdoor activities. Third, eldercare institutions should pay more attention to the mental health of older adults and should not limit their services to physical care; they can do this, for example, by setting up psychological counseling rooms. Fourth, those who make the designs of the internal environments of the eldercare institutions should consider the characteristics of the elderly as much as possible; for example, they should plan for bright light, quietness, personal privacy protection, and sufficient indoor activity space.

## Conclusion

This study suggests that the elderly living alone and those living in eldercare institutions are significantly more likely to have depressive symptoms than those living with household members. In addition, outdoor activities play a moderating role between different living arrangements and depressive symptoms. In future work, community or eldercare institution staff need to encourage older people, especially those living alone or in eldercare institutions, to actively participate in the outdoor activities suitable for them.

## Data availability statement

Publicly available datasets were analyzed in this study. This data can be found here: https://opendata.pku.edu.cn/dataverse/CHADS.

## Ethics statement

The studies involving human participants were reviewed and approved by Biomedical Ethics Review Committee of Peking University(IRB00001052–13074). The patients/participants provided their written informed consent to participate in this study.

## Author contributions

RX and CX designed the study and assessed the quality of the studies and wrote the first draft of the manuscript. YL and RX conducted data analysis. CX monitored article quality and language polish. YL, YY, and TM extracted the data from CLHLS according to the inclusion criteria and proofread these data. All authors contributed to and have approved the final manuscript.

## Conflict of interest

The authors declare that the research was conducted in the absence of any commercial or financial relationships that could be construed as a potential conflict of interest.

## Publisher's note

All claims expressed in this article are solely those of the authors and do not necessarily represent those of their affiliated organizations, or those of the publisher, the editors and the reviewers. Any product that may be evaluated in this article, or claim that may be made by its manufacturer, is not guaranteed or endorsed by the publisher.

## References

[1] Boccardi V. Population ageing: the need for a care revolution in a world 2 0. Geriatrics (Basel). (2019) 4:47. 10.3390/geriatrics4030047 31416277 PMC6787703

[2] National Bureau of Statistics of China. Statistical Communiqué of the People's Republic of China on National Economic and Social Development in 2021. (2022). Available online at: http://www.stats.gov.cn/xxgk/sjfb/zxfb2020/202202/t20220228_1827971.html (accessed May 20, 2022).

[3] Wu L Huang Z Pan Z. The spatiality and driving forces of population ageing in China. PLoS ONE. (2021) 16:e243559. 10.1371/journal.pone.0243559 33428682 PMC7799793

[4] Chang AY Skirbekk VF Tyrovolas S Kassebaum NJ Dieleman JL. Measuring population ageing: an analysis of the global burden of disease study 2017. Lancet Public Health. (2019) 4:e159–67. 10.1016/S2468-2667(19)30019-2 30851869 PMC6472541

[5] Li R Cheng X Schwebel DC Yang Y Ning P Cheng P. Disability-adjusted life years associated with population ageing in China, 1990-2017. BMC Geriatr. (2021) 21:369. 10.1186/s12877-021-02322-7 34134664 PMC8207592

[6] Malhi GS Mann JJ. Depression. Lancet. (2018) 392:2299–312. 10.1016/S0140-6736(18)31948-2 30396512

[7] Moussavi S Chatterji S Verdes E Tandon A Patel V Ustun B. Depression, chronic diseases, and decrements in health: results from the world health surveys. Lancet. (2007) 370:851–8. 10.1016/S0140-6736(07)61415-9 17826170

[8] Jiang C Zhu F Qin T. Relationships between chronic diseases and depression among middle-aged and elderly people in China: a prospective study from CHARLS. Current Med Sci. (2020) 40:858–70. 10.1007/s11596-020-2270-5 33123901

[9] Ma Y Xiang Q Yan C Liao H Wang J. Relationship between chronic diseases and depression: the mediating effect of pain. BMC Psychiatry. (2021) 21:436. 10.1186/s12888-021-03428-3 34488696 PMC8419946

[10] Van Orden KA Conwell Y. Issues in research on aging and suicide. Aging Ment Health. (2015) 20:240–51. 10.1080/13607863.2015.1065791 26179380 PMC4809416

[11] Zhou L Ma X Wang W. Relationship between cognitive performance and depressive symptoms in Chinese older adults: the China Health and Retirement Longitudinal Study (CHARLS). J Affect Disorders. (2021) 281:454–8. 10.1016/j.jad.2020.12.059 33360747

[12] State Council of China. The 14th Five-Year Plan for the Development of National Aging and Elderly Care Service System. (2022). Available online at: http://www.gov.cn/zhengce/content/2022-02/21/content_5674844.htm (accessed May 20, 2022).

[13] Lou M Xue Y Zhang S Dong Y Mo D Dong W. What factors influence older people's intention to enrol in nursing homes? a cross-sectional observational study in Shanghai, China. BMJ Open. (2018) 8:e21741. 10.1136/bmjopen-2018-021741 30185570 PMC6129045

[14] Xiaohua C Jing L Shuangyan W Xin L Pushiyi H Chenxi M. Mental health of the elderly under different elderly care models. Ch J Gerontol. (2018) 38:2013–4. (In Chinese).30649697

[15] Ma T. Environmental Psychology and Psychological Environment. Beijing: National Defense Industry Press. (1996).

[16] Min K Kim H Kim H Min J. Parks and green areas and the risk for depression and suicidal indicators. Int J Public Health. (2017) 62:647–56. 10.1007/s00038-017-0958-5 28337512

[17] Tao L Jiang R Zhang K Qian Z Chen P Lv Y. Light therapy in non-seasonal depression: an update meta-analysis. Psychiat Res. (2020) 291:113247. 10.1016/j.psychres.2020.113247 32622169

[18] Thompson R Fisher HL Dewa LH Hussain T Kabba Z Toledano MB. Adolescents' thoughts and feelings about the local and global environment: a qualitative interview study. Child Adol Ment H-Uk. (2022) 27:4–13. 10.1111/camh.12520 34783152

[19] Coppen A Shaw DM Malleson A. Changes in 5-hydroxytryptophan metabolism in depression. Brit J Psychiat. (1965) 111:105–7. 10.1192/bjp.111.470.105 14261721

[20] Wilson WM Marsden CA. *In vivo* measurement of extracellular serotonin in the ventral hippocampus during treadmill running. Behav Pharmacol. (1996) 7:101–4. 10.1097/00008877-199601000-00011 11224400

[21] Liu Y Hu J Liu J. Social support and depressive symptoms among adolescents during the COVID-19 pandemic: the mediating roles of loneliness and meaning in life. Front Public Health. (2022) 10:e916898. 10.3389/fpubh.2022.916898 35795697 PMC9251375

[22] Zeng Y Feng Q Hesketh T Christensen K Vaupel JW. Survival, disabilities in activities of daily living, and physical and cognitive functioning among the oldest-old in China: a cohort study. Lancet. (2017) 389:1619–29. 10.1016/S0140-6736(17)30548-2 28285816 PMC5406246

[23] Christensen K Thinggaard M Oksuzyan A Steenstrup T Andersen-Ranberg K Jeune B. Physical and cognitive functioning of people older than 90 years: a comparison of two Danish cohorts born 10 years apart. Lancet. (2013) 382:1507–13. 10.1016/S0140-6736(13)60777-1 23849796 PMC3818336

[24] Andresen EM Malmgren JA Carter WB Patrick DL. Screening for depression in well older adults: evaluation of a short form of the CES-D (Center for Epidemiologic Studies Depression Scale). Am J Prev Med. (1994) 10:77–84. 10.1016/S0749-3797(18)30622-6 8037935

[25] Huang Q Wang X Chen G. Reliability and validity of 10 - item CES - D among middle aged and older adults in China. Chin J Health Psychol. (2015) 23:1036–41. 10.13342/j.cnki.cjhp.2015.07.02324125553

[26] Huang G Duan Y Guo F Chen G. Prevalence and related influencing factors of depression symptoms among empty-nest older adults in China. Arch Gerontol Geriat. (2020) 91:104183. 10.1016/j.archger.2020.104183 32721660

[27] Jiang H Burström B Chen J Burström K. Rural–Urban inequalities in poor self-rated health, self-reported functional disabilities, and depression among Chinese older adults: evidence from the china health and retirement longitudinal study 2011 and 2015. Int J Env Res Pub He. (2021) 18:6557. 10.3390/ijerph18126557 34207132 PMC8296324

[28] Lu J Xu X Huang Y Li T Ma C Xu G. Prevalence of depressive disorders and treatment in China: a cross-sectional epidemiological study. Lancet Psychiat. (2021) 8:981–90. 10.1016/S2215-0366(21)00251-0 34559991

[29] Mu T Xu R Xu J Dong D Zhou Z Dai J. Association between self-care disability and depressive symptoms among middle-aged and elderly Chinese people. PLoS ONE. (2022) 17:e266950. 10.1371/journal.pone.0266950 35404987 PMC9000112

[30] Kim YB Lee SH. Gender Differences in the relationship between Living alone and depressive symptoms in elderly Korean adults. Iran J Public Health. (2019) 48:465–73. 10.18502/ijph.v48i3.890 31223574 PMC6570796

[31] Honjo K Tani Y Saito M Sasaki Y Kondo K Kawachi I. Living alone or with others and depressive symptoms, and effect modification by residential social cohesion among older adults in Japan: the JAGES longitudinal study. J Epidemiol. (2018) 28:315–22. 10.2188/jea.JE20170065 29398683 PMC6004365

[32] Ge L Yap CW Ong R Heng BH. Social isolation, loneliness and their relationships with depressive symptoms: a population-based study. PLoS ONE. (2017) 12:e182145. 10.1371/journal.pone.0182145 28832594 PMC5568112

[33] Ogawa K Shirai K Nozaki S Shikimoto R Sawada N Mimura M. The association between midlife living arrangement and psychiatrist-diagnosed depression in later life: who among your family members reduces the risk of depression? Transl Psychiatry. (2022) 12:156. 10.1038/s41398-022-01880-7 35410408 PMC9001692

[34] Yea M Chen Y. The influence of domestic living arrangement and neighborhood identity on mental health among urban Chinese elders. Aging Ment Health. (2014) 18:40–50. 10.1080/13607863.2013.837142 24044640

[35] Pithon KR Jesus de. Souto CS, Souza SJN, et al. Depressive symptoms and associated factors in elderly long-term care residents Sintomas depressivos e fatores associados em idosos residentes em instituição de longa permanência. Cien Saude Colet. (2019) 24:3275–82. 10.1590/1413-81232018249.30942017 31508748

[36] Ulbricht CM Hunnicutt JN Hume AL Lapane KL. Depression, anxiety, and pain among newly admitted nursing home residents. J Nurs Home Res Sci. (2019). 10.14283/jnhrs.2019.8 33748657 PMC7978416

[37] Tsai Y. Self-Care management and risk factors for depressive symptoms among elderly nursing home residents in Taiwan. J Pain Symptom Manag. (2006) 32:140–7. 10.1016/j.jpainsymman.2006.02.008 16877181

[38] Chu L Chi I. Nursing homes in China. J Am Med Dir Assoc. (2008) 9:237–43. 10.1016/j.jamda.2008.01.008 18457798

[39] Yuhuan W. Analysis for cause of the disability old people aid to Xinjiang choose long-term home care. Population Develop. (2013) 19:72–8. (In Chinese).

[40] Yang Z Jiang Y Wang M Zeng H. Current status and challenges of community-based elderly care centers in chongqing, china: a cross-sectional study. Risk Manag Healthc Policy. (2020) 13:2975–83. 10.2147/RMHP.S283145 33363421 PMC7754266

[41] Jing W. Analysis on the mental health problems of the elderly in welfare homes and the advantages of social work intervention. Soc Public Welfare. (2020) 11:169. (In Chinese).

[42] Franzen PL Buysse DJ. Sleep disturbances and depression: risk relationships for subsequent depression and therapeutic implications. Dialogues Clin Neuro. (2008) 10:473–81. 10.31887/DCNS.2008.10.4/plfranzen 19170404 PMC3108260

[43] Rautio N Filatova S Lehtiniemi H Miettunen J. Living environment and its relationship to depressive mood: a systematic review. Int J Soc Psychiatr. (2018) 64:92–103. 10.1177/0020764017744582 29212385

[44] Abdel Khalek AM Dadfar M Lester D. Death depression in Egyptian clinical and non-clinical groups. Nurs Open. (2021) 8:48–53. 10.1002/nop2.601 33318811 PMC7729647

[45] Rui C Ning R Ning G Xueni F Min L Jianxin D. Investigation and problem an analysis of nursing staff in elderly care institutions in Zhejiang province. Chin General Pract Nurs. (2019) 17:4113–7. (In Chinese).

[46] Xu Y Qi J Yang Y Wen X. The contribution of lifestyle factors to depressive symptoms: a cross-sectional study in Chinese college students. Psychiat Res. (2016) 245:243–9. 10.1016/j.psychres.2016.03.009 27565695

[47] Fortier M McFadden T Faulkner G. Evidence-based recommendations to assist adults with depression to become lifelong movers. Health Promot Chronic Dis Prev Can. (2020) 40:299–308. 10.24095/hpcdp.40.10.01 33064071 PMC7608934

[48] Wang F Lee EO Wu T Benson H Fricchione G Wang W. The effects of Tai Chi on depression, anxiety, and psychological well-being: a systematic review and meta-analysis. Int J Behav Med. (2014) 21:605–17. 10.1007/s12529-013-9351-9 24078491

[49] Yeung A Chan J Cheung JC Zou L. Qigong and Tai-Chi for mood regulation. Focus (Am Psychiatr Publ). (2018) 16:40–7. 10.1176/appi.focus.20170042 31975898 PMC6519567

[50] Chang J Chen Y Liu C Yong L Yang M Zhu W. Effect of square dance exercise on older women with mild mental disorders. Front Psychiatry. (2021) 12:699778. 10.3389/fpsyt.2021.699778 34393860 PMC8357995

