# Supplementary material for: Determining the association between different living arrangements and depressive symptoms among over-65-year-old people: The moderating role of outdoor activities
Source: Front Public Health. 2022 Aug 4;10:954416. doi: 10.3389/fpubh.2022.954416 (PMC9386358; doi:10.3389/fpubh.2022.954416)
Supplement: Supplementary file 1 [file Table_1.DOCX]

| Supplementary Table 1. Description of the basic information of the respondent. | | |  |
| --- | --- | --- | --- |
| **Category** | **Description** | **Sample size, n (%)** |  |
|  |  |  |  |
| **Dependent variable** |  |  |  |
| Depressive symptoms |  |  |  |
|  | 0= No | 9060(74.3) |  |
|  | 1= Yes | 3140(25.7) |  |
| **Independent variables** |  |  |  |
| Age |  |  |  |
|  | 0=65-80 | 5297(43.4) |  |
|  | 1=＞80 | 6903(56.6) |  |
| Gender |  |  |  |
|  | 0=Male | 5667(46.5) |  |
|  | 1= Female | 6533(53.5) |  |
| Living location |  |  |  |
|  | 0= Urban | 2871(23.5) |  |
|  | 1= Rural | 9329(76.5) |  |
| Hukou status |  |  |  |
|  | 0= Non-agricultural | 3560(29.2) |  |
|  | 1= Agricultural | 8623(70.8) |  |
| Households income |  |  |  |
|  | 0=＜10000 | 3016(25.8) |  |
|  | 1= 10000-50000 | 3721(31.8) |  |
|  | 2= 50000-100000 | 2705(23.1) |  |
|  | 3=＞100000 | 2257(19.3) |  |
| Marital status |  |  |  |
|  | 0= Married and living with spouse | 5402(44.7) |  |
|  | 1= Married and living without spouse | 224(1.9) |  |
|  | 2= Divorced | 45(0.4) |  |
|  | 3= Widowed | 6304(52.2) |  |
|  | 4= Never married | 103(0.9) |  |
| Years of schooling |  |  |  |
|  | 0= 0 | 4656(44.3) |  |
|  | 1= 1-9 | 4758(45.2) |  |
|  | 2=＞9 | 1103(10.5) |  |
| Disability |  |  |  |
|  | 0= No | 9947(81.5) |  |
|  | 1= Mild | 1341(11.0) |  |
|  | 2= Moderate | 490(4.0) |  |
|  | 3= Severe | 422(3.5) |  |
| Number of chronic diseases |  |  |  |
|  | 0= 0 | 4496(36.9) |  |
|  | 1= 1 | 4056(33.2) |  |
|  | 2= 2 | 2072(17.0) |  |
|  | 3= 3 | 928(7.6) |  |
|  | 4= ≥4 | 648(5.3) |  |
| Living arrangement |  |  |  |
|  | 0= With household members | 9747(79.9) |  |
|  | 1= Alone | 2050(16.8) |  |
|  | 2= Eldercare institution | 403(3.3) |  |
| Outdoor activities |  |  |  |
|  | 0= 0 | 3711(30.4) |  |
|  | 1= 1 | 849(7.0) |  |
|  | 2= 2 | 754(6.2) |  |
|  | 3= 3 | 2064(16.9) |  |
|  | 4= 4 | 4822(39.5) |  |
